# Sulfur bacteria promote dissolution of authigenic carbonates at marine methane seeps

**DOI:** 10.1038/s41396-021-00903-3

**Published:** 2021-02-11

**Authors:** Dalton J. Leprich, Beverly E. Flood, Peter R. Schroedl, Elizabeth Ricci, Jeffery J. Marlow, Peter R. Girguis, Jake V. Bailey

**Affiliations:** 1 Department of Earth and Environmental Sciences, University of Minnesota Twin-Cities, Minneapolis, MN 55455 USA; 2 Department of Biology, Boston University, Boston, MA 02215 USA; 3 Department of Organismic and Evolutionary Biology, Harvard University, Cambridge, MA 02138 USA

**Keywords:** Microbial ecology, Water microbiology, Biogeochemistry, Biogeochemistry, Biofilms

## Abstract

Carbonate rocks at marine methane seeps are commonly colonized by sulfur-oxidizing bacteria that co-occur with etch pits that suggest active dissolution. We show that sulfur-oxidizing bacteria are abundant on the surface of an exemplar seep carbonate collected from Del Mar East Methane Seep Field, USA. We then used bioreactors containing aragonite mineral coupons that simulate certain seep conditions to investigate plausible in situ rates of carbonate dissolution associated with sulfur-oxidizing bacteria. Bioreactors inoculated with a sulfur-oxidizing bacterial strain, *Celeribacter baekdonensis LH4*, growing on aragonite coupons induced dissolution rates in sulfidic, heterotrophic, and abiotic conditions of 1773.97 (±324.35), 152.81 (±123.27), and 272.99 (±249.96) μmol CaCO_3_ • cm^−2^ • yr^−1^, respectively. Steep gradients in pH were also measured within carbonate-attached biofilms using pH-sensitive fluorophores. Together, these results show that the production of acidic microenvironments in biofilms of sulfur-oxidizing bacteria are capable of dissolving carbonate rocks, even under well-buffered marine conditions. Our results support the hypothesis that authigenic carbonate rock dissolution driven by lithotrophic sulfur-oxidation constitutes a previously unknown carbon flux from the rock reservoir to the ocean and atmosphere.

## Introduction

The atmospheric flux of oceanic methane is mitigated though the sulfate-dependent anaerobic oxidation of methane (AOM), which serves as the dominant sink for methane in the oceans [1]. Approximately 10–20% of methane oxidized by AOM is incorporated into authigenic mineral deposits [2–5], which are estimated to account for 11–15% of the 11–15 Tmol•year^−1^ carbonate accumulation estimated for continental shelf sediments [6]. Seafloor authigenic carbonate deposits sequester carbon in what is thought to be a geologically stable phase. However, modern and ancient methane seep carbonates frequently exhibit signs of dissolution on the seafloor such as pits, holes, and micro-etchings of exposed surfaces, which have been suggested to be the result of aerobic methane oxidation, sulfur oxidation, or the combined effect of both [7–11]. These altered surfaces commonly co-occur with bacterial mats at the sediment/water interface that contain sulfur-oxidizing bacteria. Sulfur-oxidizing bacteria predominantly oxidize hydrogen sulfide (H_2_S) or thiosulfate (S_2_O_3_^2-^) completely to sulfate (SO_4_^2-^), or to intermediates that can be further oxidized to sulfate, although other possibilities exist [11–13]. Complete sulfide oxidation to sulfate with O_2_ is an acid-producing reaction:

I $${\mathrm{H}}_{\mathrm{2}}{\mathrm{S}} + {\mathrm{2O}}_{\mathrm{2}} \to {\mathrm{SO}}_{\mathrm{4}}^{{\mathrm{2}} - } + {\mathrm{2H}}^ +$$

Likewise, thiosulfate oxidation to sulfate with O_2_ is an acid producing reaction that consumes, and produces, equal moles of O_2_, and H^+^, respectively, as reaction I:

II $${\mathrm{S}}_2{\mathrm{O}}_3^{2 - } + {\mathrm{H}}_2{\mathrm{O}} + 2{\mathrm{O}}_2 \to 2{\mathrm{SO}}_4^{2 - } + 2{\mathrm{H}}^ +$$

Acid production by sulfide oxidation has been shown to mediate carbonate dissolution in freshwater systems including caves and aquifers [14–16], and in the built environment [17–19]. However, the potential for sulfur oxidation to induce dissolution of carbonates in marine systems that are well-buffered against bulk acidification has not been investigated.

Through rRNA gene sequencing of mat samples collected from the Del Mar East Methane Seep Field, we show that abundant and diverse sulfur-oxidizing bacteria colonize carbonate surfaces. We then use bioreactor experiments to compare the dissolution rates of carbonate coupons under abiotic conditions with those that occur in the presence of biofilms of sulfur-oxidizing bacteria in order to estimate plausible dissolution rates in nature. pH gradients within mineral-attached biofilms were then measured using laser scanning confocal microscopy to assess a potential mechanism of dissolution in which acid generated by sulfur oxidization overcomes buffering within the biofilm microenvironment. Finally, we use our experimentally-derived dissolution rates to estimate the potential impact of seafloor weathering of carbonates by sulfur-oxidizing bacteria on global carbonate rock reservoirs.

## Materials and methods

### Del Mar methane seep carbonate community analyses

The carbonate rock sample used for microbial community analyses was collected from Del Mar East Seep, Dive SO 177, (32°54.25456764 N, 117°46.9408327 W) at a depth of 1032.06 m. The site is in the northern portion of the San Diego Trough, about 50 km west of San Diego, California. Visible features of the seep include carbonate boulders and pavements colonized by orange and white bacterial mats, possible subsurface methane hydrate (large pits and craters), clam beds, and curtains of methane bubbles [20]. For a more in-depth discussion of the Del Mar Methane Seep, please see Grupe et al., (2015). A small chunk of the rock was sealed in a Mylar bag and shipped on dry ice to the University of Minnesota (Twin Cities). Upon arrival, the carbonate sample was temporarily stored at −80 °C until sampling for DNA extractions. All tools used for sampling biomass were autoclave-sterilized prior to use, and all work was performed in a LabConco A2 biological safety cabinet. Sterile aluminum foil was placed over ice packs to provide a cold and clean working environment. Three tubes were designated for the top of the rock, and three tubes were designated for the bottom of the rock. Biomass was scraped from the respective positions and placed in tubes. Top sample tubes had a biomass weight between 76.5–97.6 mg, and bottom sample tubes had a biomass weight between 30.7–59.3 mg. DNA was extracted from the seep samples using the ZymoBIOMICS DNA miniprep kit (Zymo, Irvine, CA). Nuclease-free water from the kit was processed alongside the samples as a negative control for iTag sequence analyses. The amplification of DNA and the generation iTag libraries of the V4 hypervariable region was performed by the University of Minnesota Genomics Center as previously described [21]. The samples and negative control were sequenced on ¼ of a lane of MiSeq for paired end 2×300 bp reads. Primers and adapters were removed with Cutadapt v. 2.10 [22]. The paired end reads were processed and assembled using DADA2 v1.16.0 [23]. The maximum expected error rate was 2 and reads detected as phiX were removed prior to error detection, merging of pairs and chimera detection. Taxonomic assignment was performed using the Silva database v. 138 [24]. Bioinformatic and statistical analyses was performed using tools in PhyloSeq 1.32.0 [25]. R package Decontam 1.8.0 [26] identified contaminating amplicon sequence variants (ASVs). Thus, ASVs of the genera *Escherichia*/*Shigella*, *Haemophilus*, *Streptococcus*, and the clade Chloroflexi S085 were bioinformatically removed from analyses. ASVs with statistically different abundances between the top and bottom surfaces were detected with DESeq2 v. 1.28.0.

### Scanning electron microscopy of seep carbonates

The carbonate rock sample used for SEM imaging was collected from the Lasuen Knoll Seep (Dive SO 170, 33°23.57489996 N, 118°0.39814252 W) at a depth of 279.705 meters. A small chunk of carbonate rock with filtered bottom water and 25% vol/vol glutaraldehyde was stored at 4 °C, shipped on ice to the University of Minnesota (Twin Cities), and then stored at 4 °C. Several small pieces of the carbonate rock were broken off and placed in an 8-well plate. The carbonate pieces in the 8-well plate were rinsed, and subjected to an ethanol dehydration series as follows: 50% for 2 h, 70% over night, 80% for 15 min, 95% (x2) for 15 min, and 100% (x2) for 15 min. Carbonate samples were then subjected to critical point drying on a Tousimis Model 780 A Critical-Point-Dryer following standard procedures, followed by sputter coating 1–2 nm Iridium in a Leica ACE600 Sputter Coater. Lastly, carbonate samples were visualized at 1.0 kV on a Hitachi SU8230 Field Emission Gun Scanning Electron Microscope.

#### Continuous flow bioreactor experiments

To investigate the potential for sulfur-oxidizing bacteria to dissolve carbonate minerals, we used flow-through biofilm reactors (herein referred to as bioreactors: CDC; CBR 90–3 CDC Biofilm Reactor). Bioreactors contained eight polypropylene coupon holder rods that each accommodate three 12.7 mm diameter coupons. The lid and coupon holder rods were mounted in a 1 L glass vessel with side-arm discharge port at ~400 mL. A liquid medium (described below and Supplementary Table 1) was circulated through the bioreactor, while mixing was generated by a magnetic stir bar at 80 RPM. Sampling of the coupons was conducted aseptically by removing individual coupon holders and harvesting the coupons, while replacing the removed coupon holder with a sterile rubber plug. Experiments were 21 days in duration. On the final day of bioreactor experiments, one coupon was transferred to a treatment imaging flow cell (herein referred to as flow cell: BioSurface Technologies; Model FC 310 Treatment Imaging Flow Cell) in order to visualize and measure the in vivo pH of the biofilm. The flow cell is an autoclavable polycarbonate plastic cell with a recessed inner circle for installing coupons with biofilms grown in CDC Biofilm Reactors, and is equipped with barbed influent and effluent connectors, and topped with a coverslip for in vivo imaging.

The bioreactor medium consisted of two solutions, salt solution 1 & 2, and six additional components: vitamin solution, trace element solution, yeast extract, sodium bicarbonate, sodium thiosulfate, and sodium metasilicate. One 10 L carboy was prepared by autoclaving 5 L of salt solution 1 and one 10 L carboy was prepared by autoclaving 4.1 L (with an additional 839 mL ddH_2_O) of salt solution 2 at 121 °C for 80 min. The salt solutions were then aseptically pumped together, followed by the addition of sterile stocks of 10 mL 1000X vitamin solution, 10 mL 1000X trace element solution, 10 mL (1000 g/L) yeast extract, 11 mL 1 M NaHCO_3_, 10 mL 1 M Na_2_S_2_O_3_, and 20 mL 100 mM NaSiO_3_ through a 0.2 μM filter.

The final medium had a pH of ~7.85 and contained approximately 1.1 mM HCO_3_^-^, 1 mM S_2_O_3_^2-^, and 9.14 mM Ca^2+^, with a saturation state with respect to aragonite (Ω_aragonite_) of ~0.5, similar to that measured near the sediment/water interface in certain seep environments where carbonate dissolution was observed to occur [7, 27]. The saturation state of aragonite was calculated using CO2SYS_v2.1 [28]. Ingredients for both salt solutions and the additional components added to the medium are presented in Supplementary Table 1. Measured saturation indices throughout bioreactor experiments are presented in Supplementary Table 2.

#### Mineral preparation for bioreactor experiments

Bulk mineral specimens of aragonite were obtained from D.J. Minerals (Butte, Montana). X-ray powder diffraction (XRD) of the aragonite sample, and peak matching indicates the mineral is nearly 100% aragonite. Samples used for the bioreactor were cored with a diamond coring bit on a drill press, cut to ~1–3 mm thickness and smoothed with a diamond saw. Coupons were then scored with a diamond tipped scribe pen to assign a reference code. After scoring, coupons were placed in a muffle furnace (NEY 2-525 Series II) at 500 °C for 4 h to remove residual organic matter, followed by weighing to 0.01 mg (CAHN 29 Automatic Electrobalance) and stored in a desiccator until being mounted in coupon holder rods.

#### Assembling the bioreactor

Weighed coupons were mounted in coupon holder rods, and fastened in place with plastic screws to avoid mineral chipping, as occurred with the stock metal screws. The bioreactor lid was equipped with a bubble trap on the media inflow port, a 0.45 μm airport, and a lure-lock covered in foil for inoculating via syringe. The entire bioreactor was autoclaved at 121 °C for 45 min.

#### Microbial inoculant

*Celeribacter baekdonensis* strain LH4 was used as the pure-culture inoculant for the bioreactor experiments. *C. baekdonensis* strain LH4 is a colorless, chemolithoheterotrophic, sulfur-oxidizing bacterium, belonging to the Rhodobacterales within the Alphaproteobacteria. It was isolated from marine sediments collected from a methane seep/brine pool at Green Canyon Block 233 [29] (Lat/Long: 27° 43.4392’ N, 91° 16.7638’ W, Depth: 648 m) in the Gulf of Mexico. Strain LH4 produces acid from thiosulfate oxidation, likely via the sox pathway (*soxABCDXYZ*) [30]. Strain LH4 also contains other genes related to sulfur oxidation, including four copies of sulfide quinone oxidoreductase ORFs and three copies that encode flavocytochrome c oxidases. Strain LH4 was grown up in the final medium, centrifuged at 10,000 G for 10 min, and washed 2X. OD_590_ for experiments was on average ~0.07 (~2.3•10^8^ cells/mL based on cell counts and growth curves yielding the equation *y* = 3•10^9^(x) + 2•10^7^ where y equals cells/mL, and x equals OD_590_) with an inoculum volume of 20 mL.

#### Bioreactor experiments

Experiments were run to measure the dissolution of aragonite in a medium with a saturation state of 0.5 with respect to aragonite, similar to that measured near the sediment/water interface in seep environments [27]. Unlike in the sediments where AOM-generated alkalinity is high and carbonate actively precipitates, at the sediment/water interface previous studies have measured a notable drop in alkalinity [11, 31–36] and carbonate dissolution was observed to occur [7–9, 11, 27]. In total, six continuous -flow bioreactor experiments were run: three biotic experiments under identical conditions at 10 °C, pH ~7.85, 1.1 mM HCO_3_^-^, 1 mM S_2_O_3_^2-^, and yeast extract; two uninoculated controls run under the same conditions as above to obtain an abiotic dissolution rate in undersaturated conditions; and one biotic experiment was run under identical conditions, minus the addition of S_2_O_3_^2-^ to elucidate the impact of heterotrophy on mineral stability.

10 L carboys containing the final medium and sterile bioreactors were assembled aseptically in a class A2 biological safety bench. Approximately 325 mL of the media was pumped into the bioreactor, followed by inoculating with 20 mL of pure culture and ~600 μL 1 M HCO_3_^-^. The luer-lock was removed and a 0.2 μM filter was added in its place. Bioreactors were then placed in a 10 °C refrigerator on a stir plate at 80 RPM.

Inoculated bioreactors were run in batch phase for 4 h to promote ample attachment of cells to aragonite coupon surfaces. Following the batch phase, the bioreactor was moved back to the clean hood where one coupon holder rod was removed and replaced with a sterile rubber stopper. Two coupons were placed in the incinerator at 500 °C for 4 h, followed by weighing for mass loss. The additional coupon was fixed in 4% PFA for 2 h, washed, and stained with DAPI [4,6-diamidino-2-phenylindole] for 30 min. Stained coupons were washed and mounted on coverslips (0.17 mm thickness) with DPX mountant for cell counting. After batch phase harvesting, the pump was turned on to the max flow rate (31 mL/min) and the first 100 mL out of the reactor was collected to measure pH, alkalinity, [Ca^2+^], and OD_590_. pH and alkalinity were measured using a Hanna Instruments total alkalinity mini titrator (HI-84431) and pH meter (HI 1131B) following the manufacturers protocol (Hanna Instruments, Woonsocket, RI, USA). [Ca^2+^] was measured using a Hach hardness test kit (product #2063900) following to the manufacturer’s protocol (Hach, Loveland, CO, USA). OD_590_ was measured on a Thermo Scientific Spectronic 20D + (Thermo Fisher Scientific, Waltham, MA, USA), where 1 mL of sterile medium was added to a cuvette to blank the spectrophotometer, and then 1 mL of the collected outflow was placed in a cuvette and measured. The pump was kept at the max flow rate for approximately 3–4 h after batch phase to remove planktonic cells and the additional HCO_3_^-^ added during batch phase. Directly after flushing the bioreactor, water chemistry measurements were taken as they were above, and then the flow rate was dropped to 10 mL/min. Henceforth, the flow rate was only adjusted to keep the bulk fluid at approximately the pH and alkalinity of the starting conditions (max flow rate of 21 mL/min during week 3). To ensure consistency of the bulk fluid chemistry, pH and alkalinity measurements were taken twice daily (8–12 h intervals), just before changing carboys. Carboys were changed about every 8–12 h, depending on the current flow rate. To change carboys, the following steps were taken: (1) The pump was briefly turned off, and sterile aluminum foil was wrapped around the outflow tubing. (2) The bioreactor and currently connected carboy were moved to the biological safety bench on a roll cart. (3) Tubing that connected the carboy to the bioreactor was disconnected, and the male-port of the tubing still connected to the bioreactor was submerged in sterile 70% EtOH. (4) A new 10 L carboy containing the final medium was removed from the 10 °C refrigerator. (5) Aluminum foil covering the female-port of the tubing on the carboy was removed, and sprayed with sterile 70% EtOH. (6) Tubing from the bioreactor and carboy were then connected and placed on the roll cart, and moved back to the 10 °C refrigerator. (7) The pump was turned back on and flow resumed. One coupon holder rod was harvested every four days for 21 days, where two coupons were incinerated and weighed for mass loss and one coupon was fixed and stained for cell counting as described above. Surface area of aragonite coupons were estimated using the diameter of the coupon, the density of aragonite (2.93 g/cm^3^), and the mass of the coupon before and after bioreactor experiments. Density and mass were used to estimate the volume of the coupon, followed by deriving an estimated surface area. Using the mass of coupons before and after experiments, we calculated the total number of moles lost per unit area. Averaging these data out over time we were able to determine a dissolution rate in units of μmol CaCO_3_ • cm^−2^ • hr^−1^, which we then converted to μmol CaCO_3_ • cm^−2^ • yr^−1^ (8,760 h/year).

#### Confocal microscopy

We utilized the flow cell in conjunction with the ratiometric dye C-SNAFL-1 [5-[6]-carboxyseminaphthofluorescein] and Hoechst 33342 for measuring the in vivo pH of 21-day-old biofilms, and visualizing the biofilm, respectively. Coupons transferred to the flow cell were connected to a peristaltic pump at 3 mL/min until imaging occurred.

#### Spectrofluorometric assays

To calibrate C-SNAFL-1 photon excitations and to evaluate the dye’s potential use in measuring pH in biofilms, 1 mL aliquots of the final media with 10 mM HEPES buffer were adjusted to pH 5.0, 5.2, 5.6, 6.0, 6.4, 6.8, 7.2, 7.6, and 8.0. The final probe concentration was ~1.09 μM of C-SNAFL-1. Additional calibrations were performed with the addition of 4% (vol/vol) *C. baekdonensis* strain LH4.

IV $${\mathrm{Ratio}} = \left( {{\mathrm{Ex}}_{488} - {\mathrm{Ex}}_{488{\mathrm{,bkgd}}}} \right)/\left( {{\mathrm{Ex}}_{561} - {\mathrm{Ex}}_{561{\mathrm{,bkgd}}}} \right)$$

#### Confocal scanning laser microscopy

All biofilm images, pH and ratio calibrations in the flow cell were collected using a Nikon TiE inverted microscope equipped with an A1Rsi confocal scan head (Nikon). Hoechst 33342 fluorescence was excited with a 405 nm laser and collected between 425 and 475 nm using a regular PMT to visualize the biofilm. SNAFL emission was collected between 570 and 620 nm with a GaAsP PMT alternating excitation with the 488 and 561 nm lasers to obtain a ratiometric image. This image was used to calculate the pH across the field (below). 10X/0.45 PlanApo and 20X/0.75 PlanApo VC objectives were used to collect 512- by 512-bit resolution z-stacks. Depths in this paper are the distance from the substratum (base of biofilm) to the focal plane (distance furthest from the biofilm).

To visualize the biofilms, flow was suspended and coupons were stained with Hoechst 33342 (2 drops/mL medium) for 15 min and then flushed. C-SNAFL-1 was added to the medium at a final concentration of 20 μM. Flow resumed with C-SNAFL-1 in the medium and then was suspended for no more than 15 min to minimize the accumulation of acidic metabolites, which could artificially modify the local pH. Image planes were set up to visualize the coupon-attached biofilm and the bulk media adjacent to the coupon in the same image. Biofilms were excited at 405 nm, and emission was detected between 425 and 475 nm. pH microenvironment images were captured using dual excitation at 488 nm and 561 nm, and single emission at 600 nm. To determine the z-stack image parameters, we first excited the sample at 405 nm to visualize the base of the biofilm, and then switched to the dual excitation channels to determine the end of the focal plane (e.g. the coverslip; point furthest away from the biofilm in the z-dimension where the signal diminishes). After determining the z-distance to image, we imaged the biofilm at 405 nm, and then the pH of the microenvironment at 488/561 nm. The pH of the microenvironment was determined by calculating the ratio of excitation intensities (i.e., pixel values) between the two channels (Ex_488 nm_/ Ex_561 nm_). All intensities were determined using NIS Elements software.

Calibration standards for CSLM were performed in the flow cell with a sterile aragonite coupon and media with 20 μM C-SNAFL-1 and 10 mM HEPES buffer at pH 8.0, 7.8, 7.7, 7.55, 7.2, 7.0, and 6.8. Calibrations were performed with identical microscope settings as in the experiment above. Titration curves of pH versus ratios were calculated using NIS Elements software according to Eq. IV and were used to convert C-SNAFL-1 excitation ratios to pH values.

#### Image analysis

pH gradients within the biofilm and surrounding microenvironment were evaluated with NIS Elements software by using kymographs on the ratio images (Ex_488 nm_/ Ex_561 nm_). Each kymograph was ~500 μm in length (xy) and ~100 μm in depth (z). The resulting kymograph image had 4–5 regions of interest selected, including 2–3 spanning the biofilm into the bulk fluid directly above, and 2-3 in the bulk fluid adjacent to the biofilm. Regions of interest varied in z-depth depending on the thickness of the biofilm, though all were 13 μm in width (xy).

### Estimation of CaCO_3_ dissolved from various seep locations

#### Seep locations

PDC1 – Dive, SO 162; Site, Point Dume; Feature, Chimney complex 1; Latitude, 33 56.45753693; Longitude, 118 50.70879299; Depth (m), 729.522

PDC2 – Dive, SO 164; Site, Point Dume; Feature, Chimney complex 2; Latitude, 33 56.50074983; Longitude, 118 50.64165898; Depth (m), 725.723

PDC3 – Dive, SO 164; Site, Point Dume; Feature, Chimney complex 3; Latitude, 33 56.45417243; Longitude, 118 50.70728425; Depth (m), 728.966

#### Determining total surface area

Each image taken by submersible at Point Dume had two scale bars generated at the time of imaging, one in the background and one in the foreground, with each representing 10 cm. These two scale bars, in conjunction with a designed macro in Fiji/ImageJ were used to interpolate the total surface area within each image. The macro is based on the equation *y-y*_*1*_ = *m(x-x*_*1*_*)*. The slope, *m*, was determined using the equation *((y*_*2*_*-y*_*1*_*)*/*(x*_*2*_*-x*_*1*_*))*, where *y*_*2*_ = *10* *cm/(length in pixels of the foreground scale bar), y*_*1*_ = *10* *cm/(length in pixels of the distant scale bar), x*_*2*_ = *(y-coordinate of the foreground scale bar), x*_*1*_ = *(y-coordinate of the distant scale bar)*. Each image was imported into Fiji/ImageJ, and scaled to 1333 × 750 pixels. All scales were removed to put all measurements in pixel values. Centroid and integrated density were added to set measurements. Each image was duplicated and the duplicated images were changed to 32-bit images. The macro was then run on each respective image. A polygon was then drawn around the visible area within each image, and added to the ROI manager. The ROIs were added to their respective duplicate and measured. These data gave us the total visible surface area present in each image.

#### Determining surface area of exposed carbonates and carbonated-attached bacterial mats

Polygons were drawn around all exposed carbonates within the visible region. These ROIs were then added to their respective duplicated image, and the total area of exposed carbonates was measured. Carbonate ROIs that contained small regions in which bacterial mats were not visible had “negative” ROIs drawn around them. When applicable, these negative ROIs were subtracted from the total exposed carbonate measurement, resulting in the total mat-on-rock surface area measurement. It is likely that bacteria are also colonizing the surfaces on which no obvious mat can be observed, but removal of these areas from consideration was done to provide a conservative lower estimate of bacterial coverage.

#### Moles of carbonate rock dissolved was calculated as follows

Experimentally-derived carbonate dissolution rate = 1773.97 μmol CaCO_3_ • cm^−2^ • yr^−1^.

Apply dissolution rate to seep surface area covered by rock (e.g. Hydrate Ridge) to obtain the annual dissolution; 17.7397 mol CaCO_3_ • m^−2^ • yr^−1^ * 3.00E + 05 m^2^ = 5.32E + 06 mol CaCO_3_ • yr^−1^.

Apply 92.77% average carbonate-attached bacterial mat percent coverage (Supplementary Table 3) to annual dissolution; (5.32E + 06 mol CaCO_3_ • yr^−1^) • (0.9277) = 4.94E + 06 mol CaCO_3_ • yr^−1^.

We then applied these calculations to the remaining five seep sites in Table 1 to obtain the annual amount of carbonate dissolved per seep.

**Table 1:** Estimation of moles of carbon potentially released to the ocean/atmosphere system from the weathering of carbonate rocks via lithotrophic sulfur-oxidation at seep sites where carbonate coverage has been estimated.

Site	Seep surface area (m^2^)	Seep surface area covered by rock (m^2^)	Proportion of seep covered by rock (%)	mol C dissolved • yr^-^	Reference
Hydrate Ridge	3.97E + 05	3.00E + 05	75.6	4.94E + 06 (± 9.03E + 05)	Teichert et al., 2005
Costa Rica Margin	4.91E + 05	2.40E + 05	48.9	3.95E + 06 (± 7.22E + 05)	Klaucke et al., 2008
Chilean Continental Margin	2.06E + 06	7.36E + 05	35.7	1.21E + 07 (± 2.21E + 06)	Klaucke et al., 2012
New Zealand Omakere Ridge	1.28E + 06	4.77E + 05	37.3	7.85E + 06 (± 1.44E + 06)	Jones et al., 2010
New Zealand Wairarapa Area	1.43E + 05	4.02E + 04	28.1	6.62E + 05 (± 1.21E + 05)	Klaucke et al., 2010
Santa Monica Basin	5.02E + 05	3.94E + 05	78.5	6.84E + 06 (± 1.19E + 06)	Paull et al., 2008

## Results and discussion

### Carbonate-attached mats contain sulfur-oxidizing bacteria

Mats of sulfur-oxidizing bacteria have been recorded at many localities worldwide including on the surface of authigenic carbonates at methane seeps [37–39]. Large sulfur-oxidizing bacteria of the family Beggiatoaceae may form conspicuous mats; while the presence of other sulfur oxidizers can be inferred from chemical profiles indicating the presence of sulfide oxidation at the sediment-water interface [40, 41]. In addition to the presence of macroscopic mats that can be observed by submersible, some previous molecular studies have also identified sulfur-oxidizing bacteria associated with submerged carbonate nodules [42–44] and carbonates exposed at the sediment-water interface [45, 46]. These studies demonstrate that sulfur-oxidizing bacteria are enriched in exposed carbonates vs. buried nodules and neighboring sediments. These prior studies analyzed the microbial communities of pulverized rock samples. Our study focused on the surface of a partial exposed carbonate rock sample collected by submersible from Del Mar East Methane Seep (Supplementary Figure 1). Our sampling method compares the top exposed surfaces vs. the bottom surfaces in contact with the sediment while excluding (auto-)endolithic community members [47]. We performed iTag sequencing of the V4 region of the 16S rRNA gene and found amplicon sequence variants (ASVs) representing diverse clades that are known to use sulfide and other reduced sulfur compounds for lithotrophic growth.

**Fig. 1:**
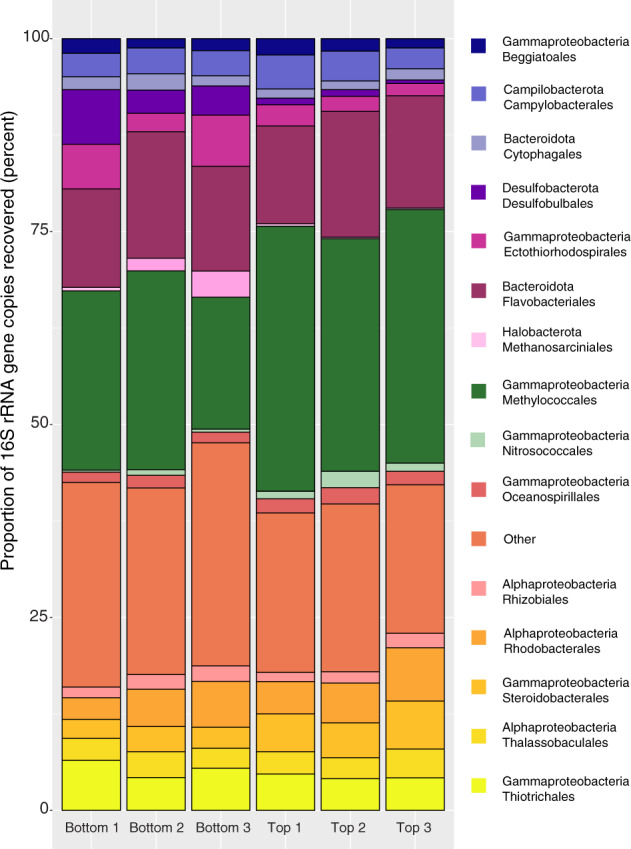
The most abundant representatives of clades on the upper vs. lower surfaces of a carbonate rock from the Del Mar methane seep. The bar plot shows representatives of clades in abundance greater than four percent of amplicon sequence variants as determined via sequencing the V4 region of the 16S  rRNA gene.

**Fig. 2:**
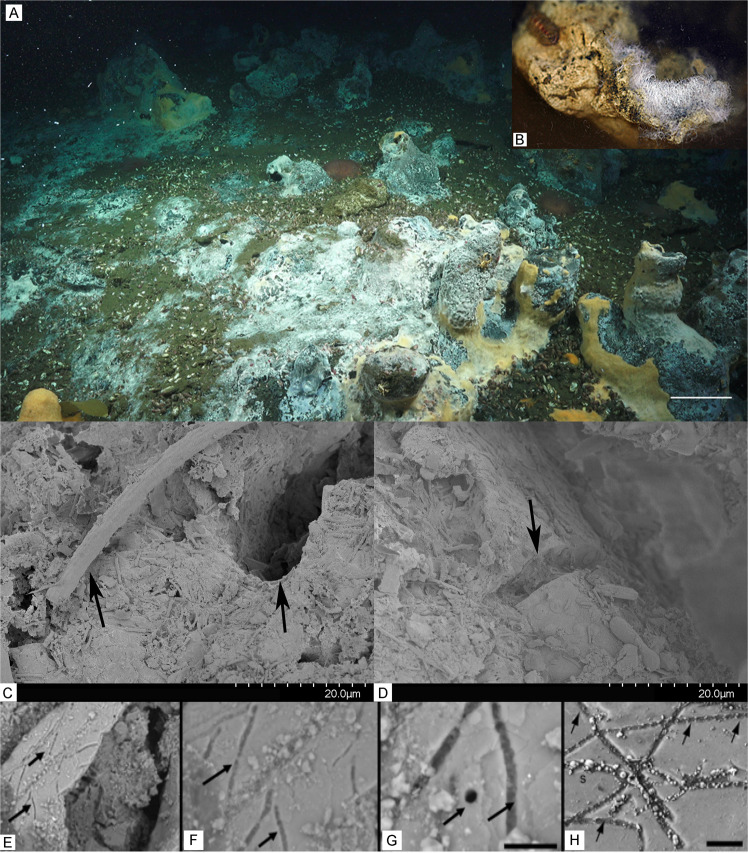
Example carbonates colonized by putative sulfur-oxidizing bacteria exhibiting dissolution features. Authigenic carbonates from Point Dume seep field are colonized by large filamentous sulfur-oxidizing bacteria, Beggiatoaceae. along with biofilms of other smaller bacteria (**A**, **C**, **D**). Pits, holes, and etchings in the carbonate surface adjacent to the cells resemble cell morphologies of the attached bacteria (**B**–**D**). SEM imaging of Costa Rica Margin seep carbonates (**E**–**G**) revealed etched surfaces with trenches and pitting of the mineral surfaces that is consistent with the size and shape of bacteria, and resemble features found in non-marine systems (**H**, from Engel et al., 2004) where carbonates are being dissolved by sulfur-oxidizing bacteria. Scale bar in **G** = 10 μm, **H** = 20 μm.

The community composition and diversity differed between the top (water exposed) and bottom (sediment exposed) surfaces of the carbonate (Fig. 1, Supplementary Figs. 2 and 3). In general, the bottom surfaces had greater diversity and richness than the top surfaces. The most striking difference between the two surfaces was greater prevalence of AOM associated microbes [42] on the bottom surface (Methanosarcina, esp. ANME-2b, and unclassified Desulfobulbaceae) and aerobic methylotrophs on the top surfaces (esp. ASVs of Marine Methylotrophic Group 2). However, both surfaces contain diverse taxa from clades that are known obligate or facultative sulfur-oxidizing bacteria, particularly those within the Gammaproteobacteria. Examples of such Gammaproteobacterial ectotypes include *Candidatus* Marithrix [48] and *Ca*. Marithioploca spp. [49] of the Beggiatoales, *Cocleimonas* spp. [50] and other Thiotrichales, *Woeseia* spp. of the Steroidobacterales [51], *Thiohalophilus* spp. [52] as well as other Ectothiorhodospirales. But other sulfur-oxidizing ectotypes were present including ASVs within the Camphylobacterales (esp. *Sulfurovum* and *Sulfurirmonas* spp.) [53] and within the Rhodobacterales, (Roseobacteria clade NAC1 [54] and unclassified Rhodobacteraceae). Interestingly, our model strain, *C. baekdonensis* strain LH4 a member of the Roseobacteria group, possesses only the complete Sox pathway, but some members of this clade also possess the reverse Dsr pathway making this clade metabolically versatile in their capacity to oxidize sulfide and thiosulfate [55]. The prevalence of specific ecotypes of sulfur-oxidizing bacteria likely depends on the concentration of sulfide and other nutrients such as available carbon substrates, but filamentous communities dominated by Gammaproteobacteria, esp. *Ca*. Marithrix, are proposed to be well-established mat communities vs. early successional communities dominated by the Campylobacterota (formally known as Epsilonproteobacteria) [45, 56]. By scanning electron microscopy, we also show that some putative filamentous sulfur bacteria are associated with pitting features on the rock surface (Fig. 2). Combined with previous descriptions from the literature [38, 42, 43, 57, 58], our results support the hypothesis that carbonate surfaces at methane seeps host diverse sulfur-oxidizing bacteria.

### Biofilm reactor experiments

**Fig. 3:**
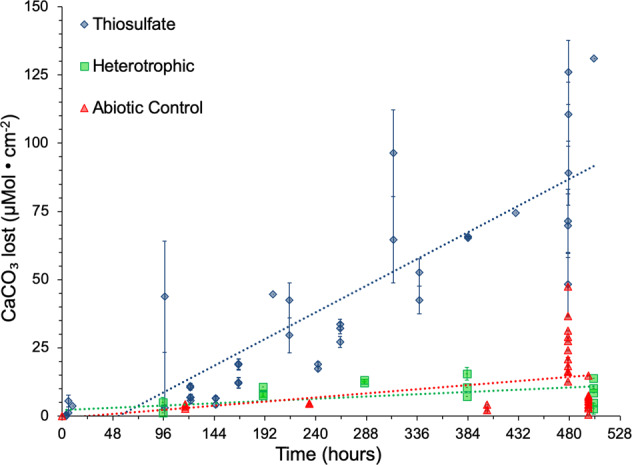
Mass loss of aragonite coupons in inoculated sulfidic and heterotrophic bioreactors, as well as uninoculated control bioreactors. Each data point represents one aragonite coupon from a bioreactor (Thiosulfate, *n* = 41; Heterotrophic, *n* = 17; Abiotic Control, *n* = 34). The mass loss (initial mass – final mass) was converted from mg to μmol CaCO_3_ • cm^-2^ and plotted against the total time the coupon was in the bioreactor. Linear regressions were performed for each test condition to extrapolate an annual dissolution rate. The line of best fit for each condition is plotted (dashed colored lines), and error bars are s.e. for the coupons from that time point.

To simulate plausible dissolution rates that could be occurring in biofilms associated with natural marine seep carbonates, we conducted a series of reactor experiments to investigate the influence of a bacterial biofilm on carbonate dissolution rates. For these experiments, we inoculated bioreactors containing carbonate rock coupons with a pure culture of *C. baekdonensis* strain LH4 [30], a chemolithoheterotrophic alphaproteobacterium that we isolated from a marine methane seep. *C. baekdonensis* LH4 is capable of lithotrophic growth via complete oxidation of S_2_O_3_^2-^ to SO_4_^2-^ + 2H^+^. It can also grow heterotrophically on yeast extract, thus we were able to separate the presence of the microbes from their metabolism and show that their metabolism is the mechanism of dissolution rather than the physical attachment. In total, six biofilm reactors were run under continuous flow for three weeks. Three independent bioreactors (designated “thiosulfate” in Fig. 3) were run with the addition of S_2_O_3_^2-^ to induce a sulfur-oxidizing metabolism that is known to generate acidity. Two independent uninoculated control bioreactors were run with the addition of S_2_O_3_^2-^ to determine the abiotic dissolution rate of aragonite. Additionally, one bioreactor was inoculated, but without S_2_O_3_^2-^ (designated “heterotrophic” in Fig. 3) to measure the influence of a heterotrophic metabolism (specifically the production of CO_2_ from organic carbon oxidation) on aragonite dissolution rates. Aragonite dissolution rates for each test condition – thiosulfate (sulfidic), heterotrophic, and abiotic control – were 1773.97 (±324.35) μmol CaCO_3_ • cm^−2^ • yr^−1^, 152.81 (±123.27) μmol CaCO_3_ • cm^−2^ • yr^−1^, and 272.99 (±249.96) μmol CaCO_3_ • cm^-2^ • yr^−1^, respectively. The bulk solutions pH (~7.75-90) and alkalinity (~1.1-3 mEq/L) of the biofilm reactors remained nearly constant for three weeks in all six reactors. However, these results show that the production of sulfuric acid by S_2_O_3_^2-^ oxidation increases the aragonite dissolution rate by nearly a factor of 12, and 7, when compared to CO_2_ production from organic carbon oxidation, and abiotic dissolution, respectively. We interpret this dissolution to primarily result from protons produced from lithotrophic sulfur-oxidation creating an acidic microenvironment, a hypothesis that we further tested by measuring the pH within the biofilm.

### Imaging biofilm acidification

Previous work in non-marine settings has demonstrated that metabolic acid production can create acidic microenvironments within a biofilm that can lead to mineral dissolution[14]. In order to test the hypothesis that lithotrophic sulfide oxidation can also create acidic microenvironments under carbonate-buffered marine conditions, we used laser scanning confocal microscopy in conjunction with a pH sensitive fluorophore, 5-[6]-carboxyseminaphthofluorescein (C-SNAFL-1), to visualize and measure the pH of the aragonite-attached biofilms.

**Fig. 4:**
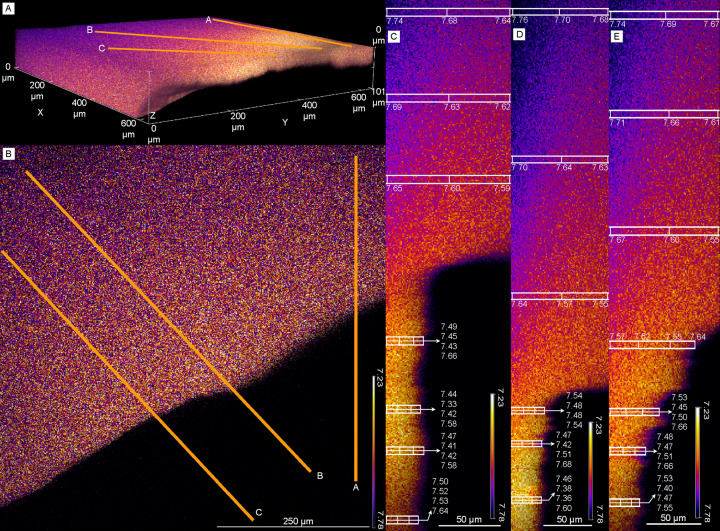
Confocal laser scanning microscopy pH imaging of biofilm attached to aragonite coupon under thiosulfate-containing conditions. C-SNAFL-1 stained C. baekdonensis LH4 biofilm growing under the presence of thiosulfate on an aragonite coupon. **A** 3D view of the aragonite coupon and bulk solution inside the flow cell. The bulk solution is colored and the coupon is the black space directly under it. Orange lines (**A**, **B**) are kymograph lines that correspond to their respective images in **C**, **D** and **E**. **B** 2D view of the aragonite coupon with orange lines showing kymograph transects. **C**–**E** kymograph profiles show a pH of ~7.65 at the biofilm-mineral interface, decreasing to a low of 7.33 within the biofilm, and increasing to ~7.50 directly above the biofilm. pH values show less variability and reach near bulk bioreactor values (7.85) moving away from the biofilm in XYZ-dimensions.

In biofilms grown in the presence of S_2_O_3_^2-^ we observed a pH gradient starting at the mineral surface, progressing through the biofilm and into the medium above (Fig. 4). At the mineral surface, the site of microbial attachment, the pH ranged from 7.68-7.54 (Fig. 4C–E). Within the biofilm the pH decreased to the lowest observed value of 7.33 (Fig. 4D). Just outside of the biofilm and in the bulk solution, the pH increased to 7.44-57 (Fig. 4C–E). Adjacent to the biofilm and mineral, in the bulk solution of the flow cell, the pH increased in the xy dimensions (Fig. 4), and to an even greater extent in the xyz dimensions, reaching a maximum value of 7.76 (Fig. 4D), nearly the pH value of inflowing media (~7.80).

We interpret this pH gradient to represent the influence of the acid produced by thiosulfate oxidation produced by the following reaction:

II $${\mathrm{S}}_2{\mathrm{O}}_3^{2 - } + {\mathrm{H}}_2{\mathrm{O}} + 2{\mathrm{O}}_2 \to 2{\mathrm{SO}}_4^{2 - } + 2{\mathrm{H}}^ +$$

These protons are then neutralized by the dissolution of the aragonite mineral by the following reaction:

III $$2{\mathrm{H}}^ + + 2{\mathrm{CaCO}}_3 \to 2{\mathrm{HCO}}_3^ - + 2{\mathrm{Ca}}^{2 + }$$

Resulting in the following net reaction:

IV $$2{\mathrm{CaCO}}_3 + {\mathrm{S}}_2{\mathrm{O}}_3^{2 - } + 2{\mathrm{O}}_2 + {\mathrm{H}}_2{\mathrm{O}} \to 2{\mathrm{SO}}_4^{2 - }\\ + 2{\mathrm{HCO}}_3^ - + 2{\mathrm{Ca}}^{2 + }$$

**Fig. 5:**
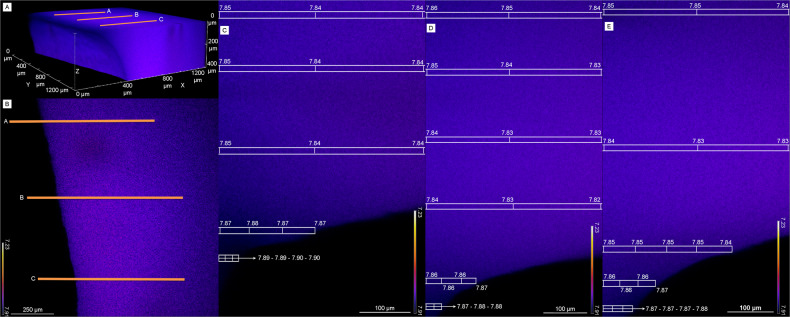
Confocal laser scanning microscopy pH imaging of biofilm attached to aragonite coupon under heterotrophic conditions. C-SNAFL-1 stained *C. baekdonensis* LH4 biofilm on aragonite coupon under heterotrophic (control) conditions. **A** 3D view of the aragonite coupon and bulk solution inside the flow cell. The bulk solution is colored and the coupon is the black space directly under it. Orange lines (**A**, **B**) are kymograph transects that correspond to their respective images in **C**, **D** and **E**. **B** 2D view of the aragonite coupon showing where kymograph lines are drawn. **C**–**E**, Kymograph profiles show no pH gradient and remain stable at the mineral surface, through the biofilm, above the biofilm, and within the bulk medium.

We believe that dissolution and subsequent proton neutralization is occurring at the biofilm-mineral interface, as this explains the pH values observed at the mineral surface. The lowest pH value recorded, 7.33, occurs in the middle of the biofilm—in the region that is most isolated from the buffering capacity of the aragonite mineral and overlying medium. The pH increase from the biofilm to the overlying medium is attributed to the large buffering capacity of the flowing medium, which is meant to simulate the practically unlimited acid-buffering capacity of the bulk ocean. We were unable to obtain pH values further away (z-dimension) from the biofilm and minerals surface due to physical limitations in the microscopy setup. However, the bulk pH values of the flow cell and bioreactor are near inflow conditions (~7.80), indicating that the acidity produced by sulfur-oxidizing bacteria is confined to the microenvironment. Under heterotrophic conditions (growth without S_2_O_3_^2-^), no pH gradient was observed (Fig. 5). Starting at the minerals surface, going through the biofilm and into the bulk media above the biofilm, the largest pH change was 0.01 units. Furthermore, in the bulk medium adjacent to the mineral and biofilm, the largest pH change was 0.02 units. In both sulfidic and heterotrophic conditions, aragonite-attached biofilms were nearly identical in thickness (Supplementary Fig. 4).

These results suggest the following: (1) oxidation of S_2_O_3_^2-^, and perhaps other reduced sulfur species, leads to the production of sulfuric acid that can dissolve aragonite even under buffered marine conditions; and (2) acidity generated by oxidizing S_2_O_3_^2-^ is greatest near the mineral surface, and is largely confined to that microenvironment.

### Implications for the global carbon cycle

Our bioreactor experiments and observations of seep samples reveal the potential for sulfur-oxidizing bacteria to contribute to carbon flux from the carbonate rock reservoir to the ocean and atmospheric carbon reservoirs.

**Fig. 6:**
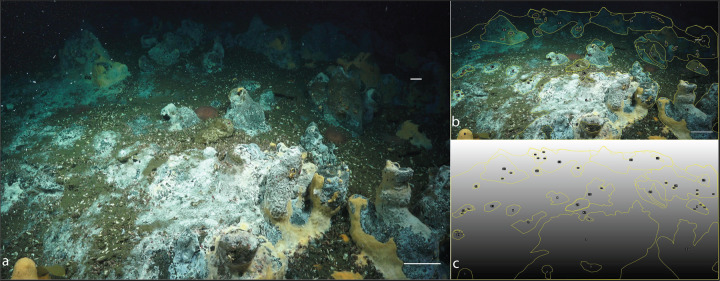
Estimating coverage of carbonates and biofilms from Point Dume Complex 3 (PDC3). Biofilms of sulfur-oxidizing bacteria colonize seep carbonates. Scale bars in **a** are both 10 cm. The scale bars were used to generate a macro (**c**) that corrects for perspective and computes the area in a region of interest (ROI). ROIs are outlined in yellow (**b**, **c**). The total visible area was calculated first by creating an ROI around the visible area of the photo. ROIs were drawn around all exposed carbonates to generate the total rock coverage. Smaller ROIs were drawn within larger ROIs where biofilms are not present and then subtracted from the total rock coverage to generate total biofilm coverage. Area measurements were converted from cm^2^ to m^2^, and then converted to percent coverage (mat:rock, mat:total area, rock:total area).

Using existing data on authigenic carbonate rock surface coverage at cold seeps, as well as ground-truth images from the Point Dume cold seep and sulfur-oxidation-induced carbonate dissolution rates generated from our experiments, we calculated an initial estimate of the amount of carbon that can be dissolved in various well-characterized cold seeps. Using multibeam, sidescan sonar, and ground-truth images of the seafloor, six previously published site descriptions [57–62] were used to estimate the percent coverage of rocks relative to the size of the active seep. Three ground-truth images from Point Dume—designated PDC1, PDC2, and PDC3 (Supplementary Figs. 5 and 6, and Fig. 6, respectively)—were used to estimate the area and percent coverage of microbial mats on exposed carbonates at these sites. These three sites comprise ~43 m^2^ in total area, with ~25 m^2^ of exposed carbonates, or ~57% rock:total area-coverage (Supplementary Table 3). Bacterial mats attached to exposed carbonates total ~23 m^2^, or ~93% mat:rock coverage (Supplementary Table 3). While the exact percent coverage of bacterial mats on exposed carbonates at the six sites used to make these estimations are unknown, all sites have reported extensive bacterial mat coverage throughout the active seep [45, 57–63]. In this context, we ascribed the bacterial mat percent coverage calculated in the PDC images to each of the six sites, along with the sulfur-oxidation-induced carbonate dissolution rate from this study to estimate the amount of carbon dissolved from seep carbonates at these locations (Table 1).

These initial estimates come with considerable uncertainty, such as the true extent of biofilm coverage on seep carbonates and their respective metabolisms, including in situ rates of sulfide oxidation, the total surface area of exposed carbonates, percent composition of carbonate-mineral phases, and the total number of seeps globally. While the amount of methane-derived carbonate precipitated globally is not well constrained, it has recently been estimated that 1.7 Tmol•yr^−1^ (range of 0.6-3.6 Tmol•yr^−1^) of methane-derived authigenic carbonate precipitates in diffusive settings [6], which is similar to previous estimates of 1 Tmol•yr^−1^, and 1.5 Tmol•yr^−1^ [5, 64]. Although the number of cold seeps globally is poorly constrained, thousands of cold seeps have recently been detected, with tens of thousands more predicted [5, 65, 66]. Using the estimated average microbially-mediated dissolution per cold seep from the six locations in Table 1 (based on our experimentally-derived rates) and extrapolating that to 10,000 seeps, we estimate the amount of carbonate dissolved via sulfide-oxidation to be 6.06•10^10^(± 1•10^10^) mol C•yr^−1^, or 7.28•10^8^ (±1.20•10^8^) kg C•yr^−1^. For comparison, estimated anthropogenic changes resulting in sediment-water interface calcite dissolution is 4•10^11^ (± 1•10^11^) kg C•yr^−1^ [67]. And it is important to note, that anthropogenic contribution to calcite dissolution mostly occurs in distinct “hot spots” where anthropogenic CO_2_ is introduced to bottom waters, i.e. northwestern Atlantic Ocean, and around islands in the Pacific and Indian Oceans. Marine saturation states of aragonite, which is more soluble than calcite, are already responding strongly to anthropogenic inputs of CO_2_ [68] and are projected to continue to rapidly decline, but not to the extent that most seep carbonates would be affected within this century [69].

While these estimates come with considerable uncertainty, even with lower amounts of mat coverage in some areas, these fluxes constitute an annual geological lability of 3.56% (range: 1.38–11.92%) of the methane-derived carbon that is thought to be sequestered in stable form as authigenic carbonates. Additionally, some mats are long-lived and grow up to several centimeters thick and are known to span hundreds of meters in diameter, which may allow for more acid to accumulate in the biofilm and subsequently dissolve more carbon than estimated here [39, 45, 70, 71]. It should be noted that this estimation is contingent upon carbonates being exposed at the surface where sulfur-oxidizing bacteria can colonize carbonates, and have O_2_ available as an electron acceptor. Community composition and available organic substrates likely influence dissolution rates as well. For example, thiosulfate-oxidizing chemoorganotrophs produce tetrathionate that generates hydroxide and this could act to counter acid production [72], although tetrathionate is short lived in sediments [12]. Also, intracellular sulfur globule producing sulfur-oxidizers such as *Ca*. Marithrix sequester reduced sulfur. The environmental triggers and the rates of sulfur globule oxidation to sulfuric acid are currently unknown in natural seep settings. Likewise, autotrophy, heterotrophy and fermentation are competing processes that could affect the saturation state of the carbonates through the uptake and release of inorganic carbon. The influence of these metabolisms should be considered in future to work to understand the dynamics of biofilm communities on the dissolution of carbonates. Additionally, our bioreactor experiments examine the effects of lithotrophic dissolution on aragonite, which is a major mineral phase in many seep carbonates. However, other carbonate phases present in carbonate assemblages at methane seeps are less soluble and future work will be required to determine the influence that sulfur-oxidation may have on minerals such as calcite and dolomite. These results demonstrate a novel microbially-mediated aspect of carbon cycling at methane seeps in the form of seafloor carbonate weathering mediated by lithotrophic acid production. Furthermore, they underscore the need for improved quantification of the number of methane seeps globally, the true extent of exposed carbonates in methane seeps, and the extent and composition of microbial communities that colonize these carbonates.

## Supplementary information

Supplemental Information

## Data Availability

Unaltered FASTQ files are deposited in the NCBI Sequence Read Archive, Bioproject PRJNA693332, and the assembled reads are available through the Data Repository of the University of Minnesota (https://conservancy.umn.edu/handle/11299/218173).

## References

[1] Marlow JJ, Steele JA, Ziebis W, Thurber AR, Levin LA, Orphan VJ (2014). Carbonate-hosted methanotrophy represents an unrecognized methane sink in the deep sea. Nat Commun.

[2] Naehr TH, Eichhubl P, Orphan VJ, Hovland M, Paull CK, Ussler W (2007). Authigenic carbonate formation at hydrocarbon seeps in continental margin sediments: a comparative study. Deep Sea Res Part II: Topical Stud Oceanogr.

[3] Eichhubl P, Greene H, Naehr T, Maher N (2000). Structural control of fluid flow: offshore fluid seepage in the Santa Barbara Basin, California. J Geochemical Exploration.

[4] Römer M, Sahling H, Pape T, dos Santos Ferreira C, Wenzhöfer F, Boetius A (2014). Methane fluxes and carbonate deposits at a cold seep area of the Central Nile Deep Sea Fan, Eastern Mediterranean Sea. Mar Geol.

[5] Sun X, Turchyn AV (2014). Significant contribution of authigenic carbonate to marine carbon burial. Nat Geosci.

[6] Akam SA, Coffin RB, Abdulla HAN, Lyons TW (2020). Dissolved inorganic carbon pump in methane-charged shallow marine sediments: state of the art and new model perspectives. Front Mar Sci.

[7] Cai WJ, Chen F, Powell EN, Walker SE, Parsons-Hubbard KM, Staff GM (2006). Preferential dissolution of carbonate shells driven by petroleum seep activity in the Gulf of Mexico. Earth Planet Sci Lett.

[8] Himmler T, Brinkmann F, Bohrmann G, Peckmann J (2011). Corrosion patterns of seep‐carbonates from the eastern Mediterranean Sea. Terra Nova.

[9] Matsumoto R (1990). Vuggy carbonate crust formed by hydrocarbon seepage on the continental shelf of Baffin Island, northeast Canada. Geochemical J.

[10] Paull CK, Hecker B, Commeau R, Freeman-Lynde RP, Neumann C, Corso WP (1984). Biological communities at the Florida escarpment resemble hydrothermal vent taxa. Science.

[11] Carson B, Kastner M, Bartlett D, Jaeger J, Jannasch H, Weinstein Y (2003). Implications of carbon flux from the Cascadia accretionary prism: results from long-term, in situ measurements at ODP Site 892B. Mar Geol.

[12] Zopfi J, Ferdelman T, Fossing H. Distribution and fate of sulfur intermediates-sulfite, tetrathionate, thiosulfate, and elemental sulfur-in marine sediments. Special Papers-Geological Society of America. 2004: 97-116.

[13] Houghton JL, Foustoukos DI, Flynn TM, Vetriani C, Bradley AS, Fike DA (2016). Thiosulfate oxidation by Thiomicrospira thermophila: metabolic flexibility in response to ambient geochemistry. Environ Microbiol.

[14] Engel AS, Stern LA, Bennett PC (2004). Microbial contributions to cave formation: new insights into sulfuric acid speleogenesis. Geology.

[15] Bennett P, Engel A, editors. Microbial contributions to karstification. Micro-organisms and Earth Systems Advances in Geomicrobiology, Society for General Microbiology (SGM) Symposium; 2005.

[16] Engel AS, Randall KW (2011). Experimental evidence for microbially mediated carbonate dissolution from the saline water zone of the Edwards Aquifer, Central Texas. Geomicrobiol J.

[17] Parker C (1945). The corrosion of concrete: 1. The Isolation of a species of bacterium associated with the corrosion of concrete exposed to atmospheres containing hydrogen sulphide. Aust J Exp Biol Med Sci.

[18] Sand W, Bock E (1984). Concrete corrosion in the Hamburg sewer system. Environ Technol.

[19] Pronk J, Meulenberg R, Hazeu W, Bos P, Kuenen J (1990). Oxidation of reduced inorganic sulphur compounds by acidophilic thiobacilli. FEMS Microbiol Lett.

[20] Grupe BM, Krach ML, Pasulka AL, Maloney JM, Levin LA, Frieder CA (2015). Methane seep ecosystem functions and services from a recently discovered southern California seep. Mar Ecol.

[21] Gohl DM, Vangay P, Garbe J, MacLean A, Hauge A, Becker A (2016). Systematic improvement of amplicon marker gene methods for increased accuracy in microbiome studies. Nat Biotechnol.

[22] Martin M (2011). Cutadapt removes adapter sequences from high-throughput sequencing reads. EMBnet J.

[23] Callahan BJ, McMurdie PJ, Rosen MJ, Han AW, Johnson AJA, Holmes SP (2016). DADA2: High-resolution sample inference from Illumina amplicon data. Nat Methods.

[24] SILVA. The all-species living tree (release LTPs106), the SILVA ribosomal RNA database project. 2011.

[25] McMurdie PJ, Holmes S (2013). phyloseq: an R package for reproducible interactive analysis and graphics of microbiome census data. PLOS ONE.

[26] Davis NM, Proctor DM, Holmes SP, Relman DA, Callahan BJ (2018). Simple statistical identification and removal of contaminant sequences in marker-gene and metagenomics data. Microbiome.

[27] Karaca DH, C Wallmann K (2010). Controls on authigenic carbonate precipitation at cold seeps along the convergent margin off Costa Rica Geochemistry. Geophysics, Geosystems.

[28] Pierrot D, Lewis E, Wallace D. MS Excel Program Developed for CO2 System Calculations. ORNL/CDIAC-105a. Carbon Dioxide Information Analysis Center, Oak Ridge National Laboratory, U.S. Department of Energy, Oak Ridge, Tennessee. 2006.

[29] Joye SB, MacDonald IR, Montoya JP, Peccini M (2005). Geophysical and geochemical signatures of Gulf of Mexico seafloor brines. Biogeosciences.

[30] Flood BE, Leprich DJ, Bailey JV (2018). Complete genome sequence of Celeribacter baekdonensis strain LH4, a thiosulfate-oxidizing alphaproteobacterial isolate from Gulf of Mexico continental slope sediments. Genome Announcment.

[31] Aloisi G, Wallmann K, Bollwerk SM, Derkachev A, Bohrmann G, Suess E (2004). The effect of dissolved barium on biogeochemical processes at cold seeps. Geochimica et Cosmochimica Acta.

[32] Claypool GE, Milkov AV, Lee YJ, Torres ME, Borowski WS, Tomaru H Microbial methane generation and gas transport in shallow sediments of an accretionary complex, southern Hydrate Ridge (ODP Leg 204), offshore Oregon, USA. Proceedings of the Ocean Drilling Program: Scientific Results. 2006; 204.

[33] Gieskes J, Mahn C, Day S, Martin JB, Greinert J, Rathburn T (2005). A study of the chemistry of pore fluids and authigenic carbonates in methane seep environments: Kodiak Trench, Hydrate Ridge, Monterey Bay, and Eel River Basin. Chem Geol.

[34] Himmler T, Haley BA, Torres ME, Klinkhammer GP, Bohrmann G, Peckmann J (2013). Rare earth element geochemistry in cold-seep pore waters of Hydrate Ridge, northeast Pacific Ocean. Geo-Mar Lett.

[35] Sommer S, Pfannkuche O, Linke P, Luff R, Greinert J, Drews M, et al. Efficiency of the benthic filter: Biological control of the emission of dissolved methane from sediments containing shallow gas hydrates at Hydrate Ridge. Global Biogeochemical Cycles. 2006; 20.

[36] Valentine DL, Kastner M, Wardlaw GD, Wang X, Purdy A, Bartlett DH. Biogeochemical investigations of marine methane seeps, Hydrate Ridge, Oregon. Journal of Geophysical Research: Biogeosciences. 2005; 110.

[37] Brazelton WJ, Schrenk MO, Kelley DS, Baross JA (2006). Methane-and sulfur-metabolizing microbial communities dominate the Lost City hydrothermal field ecosystem. Appl Environ Microbiol.

[38] Jørgensen BB, Boetius A (2007). Feast and famine—microbial life in the deep-sea bed. Nat Rev Microbiol.

[39] Grünke S, Felden J, Lichtschlag A, Girnth AC, De Beer D, Wenzhöfer F (2011). Niche differentiation among mat‐forming, sulfide‐oxidizing bacteria at cold seeps of the Nile Deep Sea Fan (Eastern Mediterranean Sea). Geobiology.

[40] Jørgensen B (1977). Distribution of colorless sulfur bacteria (Beggiatoa spp.) in a coastal marine sediment. Mar Biol.

[41] Otte S, Kuenen JG, Nielsen LP, Paerl HW, Zopfi J, Schulz HN (1999). Nitrogen, carbon, and sulfur metabolism in natural Thioploca samples. Appl Environ Microbiol.

[42] Mason OU, Case DH, Naehr TH, Lee RW, Thomas RB, Bailey JV (2015). Comparison of archaeal and bacterial diversity in methane seep carbonate nodules and host sediments, Eel River Basin and Hydrate Ridge, USA. Microb Ecol.

[43] Marlow JJ, Steele JA, Case DH, Connon SA, Levin LA, Orphan VJ (2014). Microbial abundance and diversity patterns associated with sediments and carbonates from the methane seep environments of Hydrate Ridge. Or Front Mar Sci.

[44] Yanagawa K, Shiraishi F, Tanigawa Y, Maeda T, Mustapha NA, Owari S (2019). Endolithic microbial habitats hosted in carbonate nodules currently forming within sediment at a high methane flux site in the Sea of Japan. Geosciences.

[45] Case DH, Pasulka AL, Marlow JJ, Grupe BM, Levin LA, Orphan VJ (2015). Methane seep carbonates host distinct, diverse, and dynamic microbial assemblages. MBio.

[46] Prouty NG, Campbell PL, Close HG, Biddle JF, Beckmann S (2020). Molecular indicators of methane metabolisms at cold seeps along the United States Atlantic Margin. Chem Geol.

[47] Marlow JJ, Peckmann J, Orphan V (2015). Autoendoliths: a distinct type of rock-hosted microbial life. Geobiology.

[48] Salman-Carvalho V, Fadeev E, Joye SB, Teske A (2016). How clonal is clonal? Genome plasticity across multicellular segments of a “Candidatus Marithrix sp.” filament from sulfidic, briny seafloor sediments in the Gulf of Mexico. Front Microbiol.

[49] Salman V, Amann R, Girnth A-C, Polerecky L, Bailey JV, Høgslund S (2011). A single-cell sequencing approach to the classification of large, vacuolated sulfur bacteria. Syst Appl Microbiol.

[50] Tanaka N, Romanenko LA, Iino T, Frolova GM, Mikhailov VV (2011). *Cocleimonas flava* gen. nov., sp. nov., a gammaproteobacterium isolated from sand snail (*Umbonium costatum*). Int J Syst Evolut Microbiol.

[51] Mußmann M, Pjevac P, Krüger K, Dyksma S (2017). Genomic repertoire of the Woeseiaceae/JTB255, cosmopolitan and abundant core members of microbial communities in marine sediments. The. ISME J.

[52] Sorokin DY, Tourova TP, Bezsoudnova EY, Pol A, Muyzer G (2007). Denitrification in a binary culture and thiocyanate metabolism in Thiohalophilus thiocyanoxidans gen. nov. sp. nov. – a moderately halophilic chemolithoautotrophic sulfur-oxidizing Gammaproteobacterium from hypersaline lakes. Arch Microbiol.

[53] Waite DW, Vanwonterghem I, Rinke C, Parks DH, Zhang Y, Takai K (2017). Comparative Genomic Analysis of the Class Epsilonproteobacteria and Proposed Reclassification to Epsilonbacteraeota (phyl. nov.). Front Microbiol.

[54] Moran MA, González JM, Kiene RP (2003). Linking a bacterial taxon to sulfur cycling in the sea: studies of the marine Roseobacter Group. Geomicrobiol J.

[55] Lenk S, Moraru C, Hahnke S, Arnds J, Richter M, Kube M (2012). Roseobacter clade bacteria are abundant in coastal sediments and encode a novel combination of sulfur oxidation genes. ISME J.

[56] Patwardhan S, Foustoukos DI, Giovannelli D, Yücel M, Vetriani C (2018). Ecological succession of sulfur-oxidizing Epsilon- and Gammaproteobacteria during colonization of a shallow-water gas vent. Front Microbiol.

[57] Teichert BM, Bohrmann G, Suess E (2005). Chemoherms on Hydrate Ridge—Unique microbially-mediated carbonate build-ups growing into the water column. Palaeogeogr, Palaeoclimatol, Palaeoecol.

[58] Paull CK, Normark WR, Ussler W, Caress DW, Keaten R (2008). Association among active seafloor deformation, mound formation, and gas hydrate growth and accumulation within the seafloor of the Santa Monica Basin, offshore California. Mar Geol.

[59] Klaucke I, Masson DG, Petersen CJ, Weinrebe W, Ranero CR. Multifrequency geoacoustic imaging of fluid escape structures offshore Costa Rica: implications for the quantification of seep processes. Geochemistry, Geophysics, Geosystems. 2008; 9.

[60] Klaucke I, Weinrebe W, Linke P, Kläschen D, Bialas J (2012). Sidescan sonar imagery of widespread fossil and active cold seeps along the central Chilean continental margin. Geo-Mar Lett.

[61] Jones AT, Greinert J, Bowden D, Klaucke I, Petersen CJ, Netzeband G (2010). Acoustic and visual characterisation of methane-rich seabed seeps at Omakere Ridge on the Hikurangi Margin, New Zealand. Mar Geol.

[62] Klaucke I, Weinrebe W, Petersen CJ, Bowden D (2010). Temporal variability of gas seeps offshore New Zealand: Multi-frequency geoacoustic imaging of the Wairarapa area, Hikurangi margin. Mar Geol.

[63] Levin LA, Mendoza GF, Grupe BM (2017). Methane seepage effects on biodiversity and biological traits of macrofauna inhabiting authigenic carbonates. Deep Sea Res Part II: Topical Stud Oceanogr.

[64] Wallmann K, Aloisi G, Haeckel M, Tishchenko P, Pavlova G, Greinert J (2008). Silicate weathering in anoxic marine sediments. Geochim Cosmochim Acta.

[65] Levin LA, Baco AR, Bowden DA, Colaco A, Cordes EE, Cunha MR (2016). Hydrothermal vents and methane seeps: rethinking the sphere of influence. Front Mar Sci.

[66] Skarke A, Ruppel C, Kodis M, Brothers D, Lobecker E (2014). Widespread methane leakage from the sea floor on the northern US Atlantic margin. Nat Geosci.

[67] Sulpis O, Boudreau BP, Mucci A, Jenkins C, Trossman DS, Arbic BK (2018). Current CaCO3 dissolution at the seafloor caused by anthropogenic CO2. Proc Natl Acad Sci Usa.

[68] Friedrich T, Timmermann A, Abe-Ouchi A, Bates NR, Chikamoto MO, Church MJ (2012). Detecting regional anthropogenic trends in ocean acidification against natural variability. Nat Clim Change.

[69] Gattuso JP, Magnan A, Bille R, Cheung WW, Howes EL, Joos F (2015). Contrasting futures for ocean and society from different anthropogenic CO(2) emissions scenarios. Science.

[70] Boetius A, Wenzhöfer F (2013). Seafloor oxygen consumption fuelled by methane from cold seeps. Nat Geosci.

[71] Gundersen JK, Jorgensen BB, Larsen E, Jannasch HW (1992). Mats of giant sulphur bacteria on deep-sea sediments due to fluctuating hydrothermal flow. Nature.

[72] Teske A, Brinkhoff T, Muyzer G, Moser DP, Rethmeier J, Jannasch HW (2000). Diversity of thiosulfate-oxidizing bacteria from marine sediments and hydrothermal vents. Appl Environ Microbiol.

