# Supplementary material for: Sulfur bacteria promote dissolution of authigenic carbonates at marine methane seeps
Source: ISME J. 2021 Feb 11;15(7):2043–56. doi: 10.1038/s41396-021-00903-3 (PMC8245480; doi:10.1038/s41396-021-00903-3)
Supplement: Supplementary file 1 — Supplemental Information [file 41396_2021_903_MOESM1_ESM.docx]

**Supplementary Information**

**
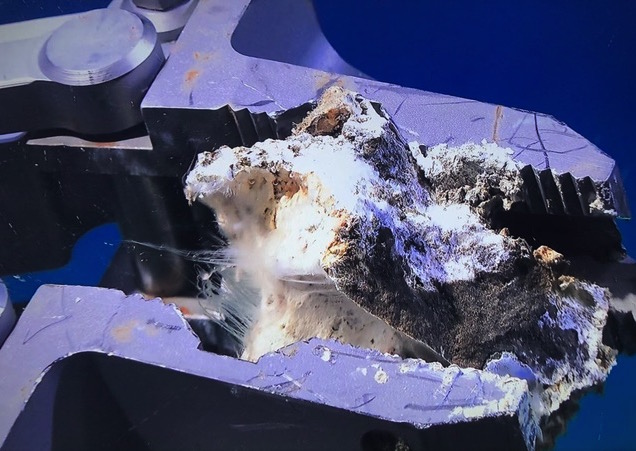
**

**Supplementary Figure 1: Carbonate rock recovered from Del Mar East seep field showing attached mats of white filamentous sulfur-oxidizing bacteria.** A portion of this rock was used for community seep and SEM analyses.

**Supplementary Figure 2: Measures of diversity of microorganisms attached to the top surfaces vs. the bottom surfaces of Point Dume carbonate.** The total number of amplicon sequence variants (ASVs) observed, as well as two measures of species richness, indicate that microbial communities attached to the bottom surfaces of the carbonate rock sampled from Point Dume, are more diverse than the top surfaces of the rock.

**Supplementary Figure 3: Rarefaction curves for bottom vs. top sampling of Point Dume seep carbonate.** Rarefaction curves demonstrate adequately sampling depth to capture the community composition. Horizontal bar indicates the sample library with the smallest library size.

**
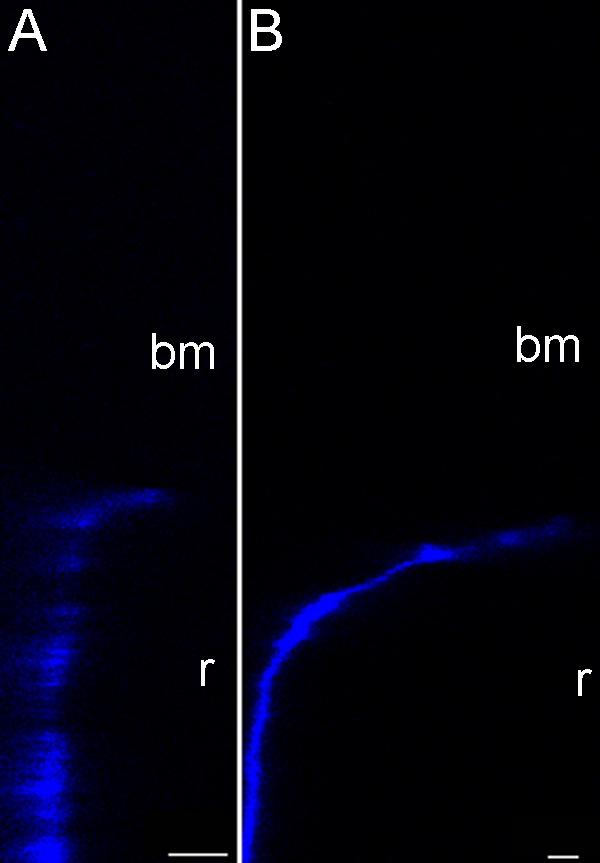
**

**Supplementary Figure 4:** Kymograph images of Hoechst 33342 stained aragonite-attached biofilms growing under sulfidic conditions (A), and heterotrophic conditions (B) using laser scanning confocal microscopy. bm = bulk medium, and r = aragonite rock. Scale bar in A and B = 25 μm.


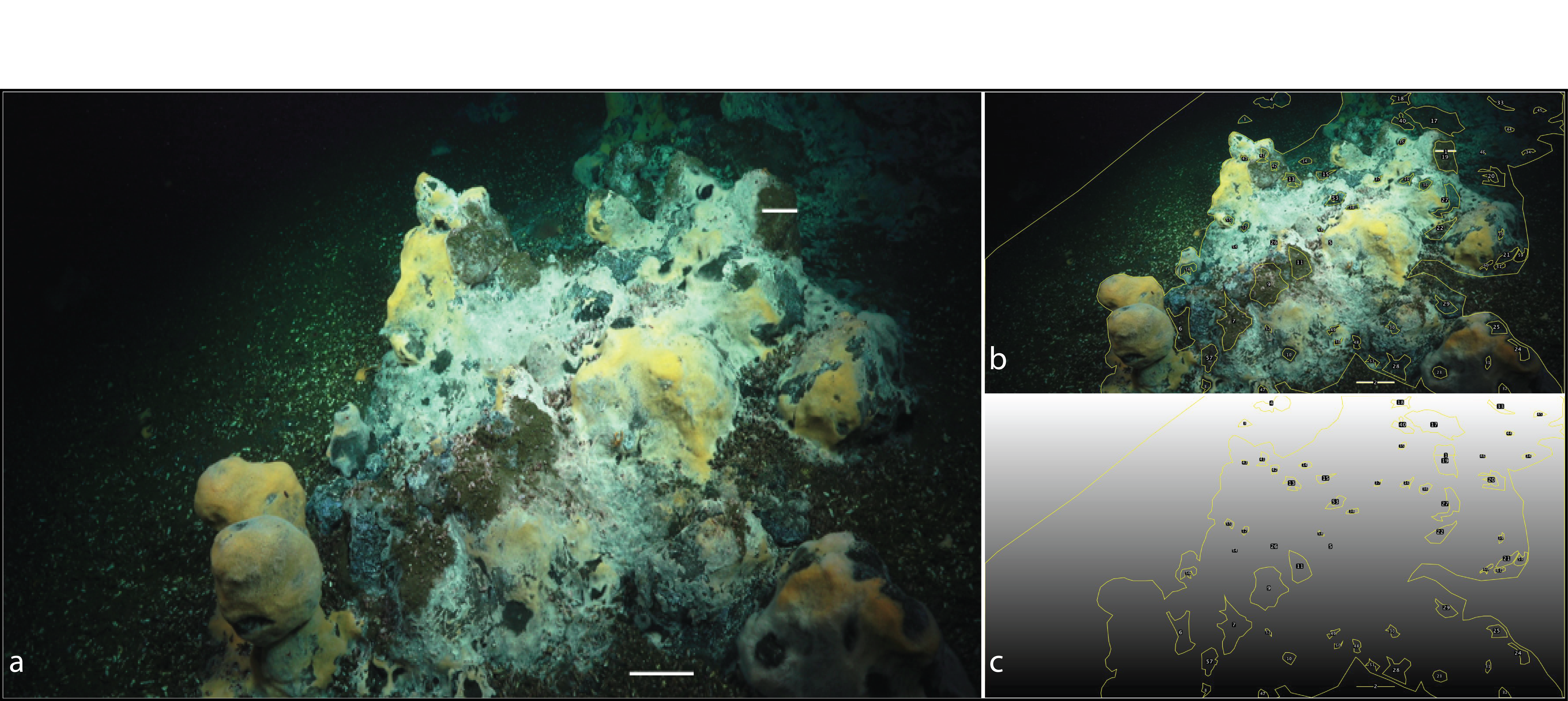


**Supplementary Figure 5: Estimating coverage of carbonates and biofilms from Point Dume Complex 3 (PDC1).** Biofilms of sulfide-oxidizing bacteria colonize seep carbonates. Scale bars in (a) are both 10 cm. The scale bars were used to generate a macro (c) that corrects for perspective and computes the area in a region of interest (ROI). ROIs are outlined in yellow (b,c). The total visible area was calculated first by creating an ROI around the visible area of the photo. ROIs were drawn around all exposed carbonates to generate the total rock coverage. Smaller ROIs were drawn within larger ROIs where biofilms are not present and then subtracted from the total rock coverage to generate total biofilm coverage. Area measurements were converted from cm^2^ to m^2^, and then converted to percent coverage (mat:rock, mat:total area, rock:total area).


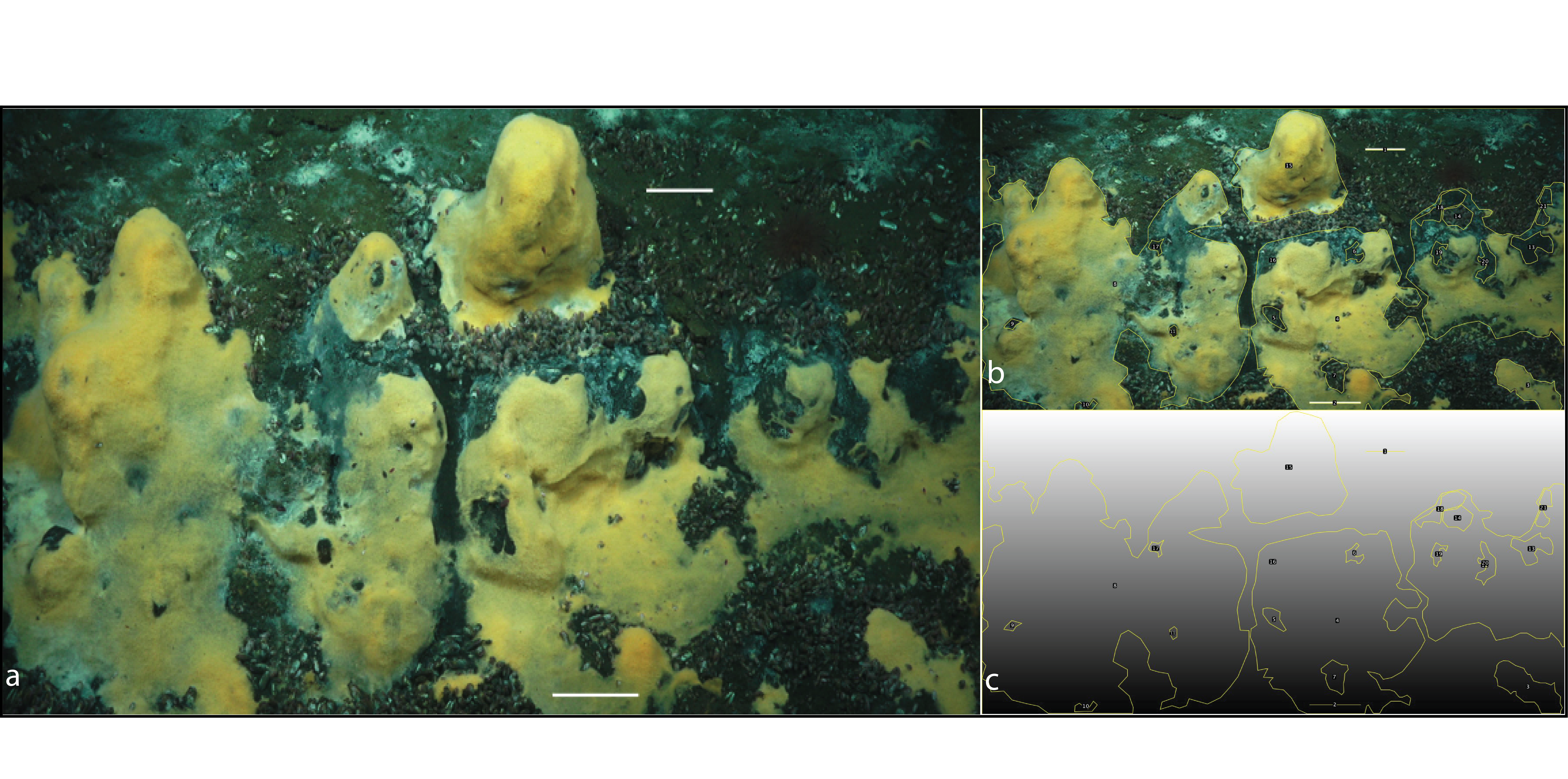


**Supplementary Figure 6: Estimating coverage of carbonates and biofilms from Point Dume Complex 2 (PDC2).** Biofilms of sulfide-oxidizing bacteria colonize seep carbonates. Scale bars in (a) are both 10 cm. The scale bars were used to generate a macro (c) that corrects for perspective and computes the area in a region of interest (ROI). ROIs are outlined in yellow (b,c). The total visible area was calculated first by creating an ROI around the visible area of the photo. ROIs were drawn around all exposed carbonates to generate the total rock coverage. Smaller ROIs were drawn within larger ROIs where biofilms are not present and then subtracted from the total rock coverage to generate total biofilm coverage. Area measurements were converted from cm^2^ to m^2^, and then converted to percent coverage (mat:rock, mat:total area, rock:total area).

**Supplementary Table 1:** Media components for salt solution I & II, trace element solution, vitamin solution, sodium bicarbonate, sodium thiosulfate, and yeast extract.

**Supplementary Table 2:** Average saturation indices during bioreactor experiments under sulfidic (thiosulfate), heterotrophic, and control conditions. Saturation values were calculated using CO2Sys_v2.1. Mean and 95% confidence values were obtained using all measured values from 3 thiosulfate bioreactors (n=91), 1 heterotrophic bioreactor (n=26), and 2 control bioreactors (n=45).

**Supplementary Table 3:** Measurements of total surface area, exposed carbonate surface area, and carbonate-attached bacterial mat surface area measured from Point Dume Seep images.

|  | PDC1 | PDC2 | PDC3 | Total |
| --- | --- | --- | --- | --- |
| Total mat on rocks (m^2^) | 8.32 | 5.47 | 8.98 | 22.78 |
| Total rock (m^2^) | 9.20 | 5.71 | 9.65 | 24.55 |
| Total area (m^2^) | 14.69 | 10.02 | 18.56 | 43.27 |
| Percent coverage (mat:rock) | 90.42 | 95.92 | 93.14 | 92.77 |
| Percent coverage (rock:total area) | 62.64 | 56.94 | 51.98 | 56.74 |
| Percent coverage (mat:total area) | 56.63 | 54.61 | 48.41 | 52.64 |
